# Validation of a second-generation appropriateness classification system for total knee arthroplasty: a prospective cohort study

**DOI:** 10.1186/s13018-021-02371-z

**Published:** 2021-03-29

**Authors:** Antonio Escobar, Amaia Bilbao, Maria L. Bertrand, Jesús Moreta, Miquel A. Froufe, Jordi Colomina, Olga Martınez-Cruz, Robert A. Perera, Daniel L. Riddle

**Affiliations:** 1 Osakidetza Basque Health Service, Basurto University Hospital, Research Unit, Bilbao, Spain; 2 Health Service Research Network on Chronic Diseases (REDISSEC), Bilbao, Spain; 3 Kronikgune Institute for Health Services Research, Barakaldo, Spain; 4 University of Malaga, Malaga, Spain; 5 Department of Orthopaedic Surgery and Traumatology, Hospital Costa del Sol, Marbella, Spain; 6 Biocruces-Bizkaia Health Research Institute, Group of Lower Limb Reconstructive Surgery, Barakaldo, Spain; 7 Osakidetza Basque Health Service, Department of Orthopaedic Surgery and Traumatology, Galdakao-Usansolo University Hospital, Galdakao, Spain; 8 Department of Orthopaedic Surgery and Traumatology, Hospital Universitari de Girona Dr Josep Trueta, Girona, Spain; 9 Department of Orthopaedic Surgery and Traumatology, Santa Maria University Hospital, Lleida, Spain; 10 Àmbit d’Avaluació, Agència de Qualitat i Avaluacio´ Sanitaries de Catalunya (AQuAS), Departament de Salut - Generalitat de Catalunya, Barcelona, Spain; 11 Department of Biostatistics, Virginia Commonwealth University, Richmond, VA USA; 12 Departments of Physical Therapy, Orthopaedic Surgery and Rheumatology, Virginia Commonwealth University, Richmond, VA USA

**Keywords:** Osteoarthritis, Knee, Surgery, Quality of life, Outcome measures, Appropriateness

## Abstract

**Background:**

To test the validity of a second-generation appropriateness system in a cohort of patients undergoing total knee arthroplasty (TKA).

**Methods:**

We applied the RAND/UCLA Appropriateness Method to derive our second-generation system and conducted a prospective study of patients diagnosed with knee osteoarthritis in eight public hospitals in Spain. Main outcome questionnaires were the Western Ontario and McMaster Universities Osteoarthritis Index (WOMAC), Short-Form-12 (SF-12), and the Knee Society Score satisfaction scale (KSS), completed before and 6 months after TKA. Baseline, changes from baseline to 6 months (journey outcome), and 6-month scores (destination outcome) were compared according to appropriateness category. Percentage of patients attaining the minimal clinically important difference (MCID) and responders according to Outcome Measures in Rheumatology-Osteoarthritis Research Society (OMERACT-OARSI) criteria were also reported.

**Results:**

A total of 282 patients completed baseline and 6-month questionnaires. Of these, 142 (50.4%) were classified as Appropriate, 90 (31.9%) as Uncertain, and 50 (17.7%) as Inappropriate. Patients classified as Appropriate had worse preoperative pain, function, and satisfaction (*p* < 0.001) and had greater improvements (i.e., journey scores) than those classified as Inappropriate (*p* < 0.001). At 6 months, destination scores for pain, function, or satisfaction were not significantly different across appropriateness categories. The percentage of patients meeting responder criteria (*p* < 0.001) and attaining MCID was statistically higher in Appropriate versus Inappropriate groups in pain (*p* = 0.04) and function (*p* = 0.004).

**Conclusions:**

The validity of our second-generation appropriateness system was generally supported. The findings highlight a critical issue in TKA healthcare: whether TKA appropriateness should be driven by the extent of improvement, by patient final state, or by both.

**Supplementary Information:**

The online version contains supplementary material available at 10.1186/s13018-021-02371-z.

## Background

Utilization of total knee arthroplasty (TKA) has risen substantially over the past few decades. In the USA in 2014, for example, 723,000 TKA surgeries were done and based on 2000–2014 data, the projected growth of 85% to 1.26 million procedures is expected by 2030 [1]. Consistent with variability in TKA utilization, there also is variability in recommendations for who should qualify for TKA [2]. Variation in TKA recommendations is important because about 20% of patients have a poor outcome after TKA [3].

Although it is generally accepted that TKA is an effective treatment for symptomatic knee osteoarthritis (OA), there are controversies about the indication criteria. There have been several attempts to establish criteria to recommend TKA, from the first studies [4, 5] to more recent works reflecting perspectives from orthopedic surgeons [6, 7], patients [8], and other stakeholders [9]. On the other hand, we found no studies applying these criteria to patients other than our first-generation appropriateness classification system for TKA [10]. This landmark paper, published in *The Journal of Evaluation in Clinical Practice* in 2003, lacked specificity with a variety of criteria (e.g., the symptomatology criteria did not have mutually exclusive categories), was developed two decades ago and is out-dated, and did not include variables related to psychological distress or comorbidity [11, 12].

It is well known that randomized clinical trials (RCTs) are the best way to assess healthcare interventions, but are lacking in TKA [13]. One alternative to the RCT is to synthesize the opinions of experts [14]. The RAND/UCLA Appropriateness Method (RUAM) [14] has been used to evaluate appropriateness in several diagnostic and surgical procedures [15, 16]. The RUAM is a consensus-based multi-step method that requires an expert panel to synthesize the evidence, identify key indicators (predictor variables) for the problem of interest, and then write a complete set of brief clinical scenarios that capture all permutations of the indicators. An independent second expert panel of experts then scores each of the scenarios using a well-established appropriateness ranking system [14]. More recently, the American Academy of Orthopaedic Surgeons (AAOS) have used RUAM to develop appropriateness criteria for several procedures including TKA [17].

We designed our study to fill an important gap in the translation of knee arthroplasty research [18] evidence by developing and testing a RAND-based classification system for knee arthroplasty appropriateness that was grounded in contemporary practice. For example, our newly proposed system includes psychological distress and comorbidity indicators that have not been used in other RAND-based methods.

Our objective was to test the validity of our second-generation RUAM-based TKA appropriateness system. Validity, in this context, is consistent with methods for testing validity and advocated by developers of the RUAM system [14]. We judged the presence of validity by comparing baseline, change, and final outcome scores of persons classified as appropriate, inappropriate, or uncertain. Outcome scores can be compared in a retrospective fashion, by applying classification criteria to already collected patient data, much like an earlier paper [18], or to patient data collected after a system is developed, the method used in the current study. Our analytic approach and hypotheses were grounded by consideration of pain, function, and satisfaction measures along a time-based continuum from preoperative baseline to change over time (i.e., the journey) and to final destination at 6 months (i.e., the final time point) [19]. We hypothesized that (1) patients classified as Appropriate would have greater pain and worse self-reported knee-related function as well as less satisfaction with their current knee health prior to TKA as compared to those classified as Inappropriate, (2) baseline to 6-month pain and function change scores would be greater for patients classified as Appropriate as compared to persons classified as Inappropriate, and (3) 6-month pain, function, and satisfaction scores would be approximately the same for all three classification groups [18].

## Methods

### Classification system development

Criteria for developing our TKA appropriateness system were based on the method developed by the RAND Corporation and the University of California in Los Angeles (RUAM) [14]. The description of the RUAM process used to generate the TKA appropriateness system has been thoroughly described elsewhere [20] and appears in brief in the Additional file 1.

### Validity study

We carried out a prospective cohort study, which took place in eight hospitals belonging to the Spanish National Health Service. This study complies with the Declaration of Helsinki, and the corresponding Institutional Review Boards approved the study (registration ID: PI2016135, issued on 29 November 2016). All patients who agreed signed a informed consent.

Consecutive patients placed on the waiting list to undergo primary TKA with OA, between January 2017 and January 2018, were eligible for the study. Patients with psychiatric diseases were excluded because of the potential for biased or incorrect responses when filling out the questionnaires. We collected data directly from patients via questionnaires. We sent the questionnaires to patients at baseline while on the waiting list and at 6 months post-surgery. The questionnaires included sociodemographic questions, pain, function, and health-related quality of life (HRQoL) instruments. A reminder letter was sent to patients who had not replied after 15 days.

### Baseline and follow-up outcome questionnaires

The Western Ontario and McMaster Universities Osteoarthritis Index (WOMAC) questionnaire is an arthritis-specific questionnaire developed for patients with hip or knee OA. It comprises 24 items, grouped into three dimensions: pain (5 items), stiffness (2 items), and physical function (17 items). We used the Likert version with five response levels for each item. The data were standardized to a range of values from 0 (best) to 100 (worst). The WOMAC has been validated in Spanish [21].

The Short-Form-12 (SF-12), a validated HRQoL measure, was also administered and provides scores on two summary measures, the mental (MCS) and physical (PCS) component summaries. Both scales are scored from 0 (worst status) to 100 (best status) and have been validated in Spanish [22].

The satisfaction subscale of the Knee Society Clinical Rating System (KSS), a widely used self-report questionnaire in TKA, was used to measure patient satisfaction. Satisfaction questions ask patients to rate the degree of satisfaction with their pain or function while sitting, lying in bed, getting out of bed, and performing light household activities and recreational activities, each on a five-level Likert scale, from very satisfied to very dissatisfied. The five satisfaction items are summed to calculate a total satisfaction score. The Spanish version has been validated [23].

### Statistical analysis

The unit of analysis was the patient meaning that only one knee of each patient was included in the data analysis. In cases where bilateral staged TKA was performed during the study period, only the data from the first TKA was used. Descriptive statistics included frequency tables, means, and standard deviations (SD). Baseline characteristics were compared for those with versus those without follow-up data using the *t*-test for continuous variables and the chi-square or the Fisher’s exact test for categorical variables. Our focus for the key analyses was on comparisons of baseline differences, comparisons of changes over the 6-month period (i.e., the journey outcomes), and comparisons at the final 6-month time point (i.e., the destination outcomes).

The WOMAC, SF-12, and KSS scores at baseline and at 6 months and changes from baseline to 6 months were compared amongst the three appropriateness groups. We used the chi-square test, analysis of variance (ANOVA) with Scheffé post hoc test, or the non-parametric Kruskal-Wallis, when appropriate, positive changes indicating improvement following surgery.

We compared the percentage of patients exceeding the WOMAC pain and function minimal clinically important difference (MCID) amongst the three appropriateness groups, by the chi-square. Previously published MCID cut-off values by quartiles of baseline severity in WOMAC were used [24]. We also compared responders according to the definition of the OMERACT-OARSI set of responder criteria [25] amongst the three appropriateness groups.

The KSS satisfaction questions were dichotomized into satisfied (answers of very satisfied and satisfied) versus unsatisfied patients (answers of neutral, dissatisfied, and very dissatisfied). The percentages of patients in each group were compared according to appropriateness by chi-square tests. All effects were considered statistically significant at *p* < 0.05. Statistical analysis was performed using SPSS v17, and SAS for Windows statistical software, version 9.4 (SAS Institute, Inc., Carey, NC).

### Sample size

We hypothesized statistically significant differences in the percentage of patients exceeding the MCID for WOMAC pain scores between the Appropriate and the Inappropriate groups, based on the results previously reported [26], where 69% were classified as Appropriate and 13% as Inappropriate. Therefore, 35 subjects classified as Inappropriate and 185 classified as Appropriate were required to detect a statistically significant difference between the percentages of patients exceeding the MCID for pain of 50% for Inappropriate and 75% for Appropriate cases. The analysis was based on an alpha risk of 0.05 and a power of 0.8 in a bilateral contrast.

## Results

A total of 334 patients who met the inclusion criteria completed the questionnaires prior to TKA. Of these, 282 patients (84.4%) returned the questionnaires at 6 months. There were no significant differences (Table 1), among those with versus those without 6-month follow-up data regarding any variable. Of the 282 participating patients with completed data, 142 were classified as Appropriate (50.4%), 90 as Uncertain (31.9%), and 50 as Inappropriate (17.7%). The mean age was 70.9 years (SD, 8.3) and 184 (65.2%) were women. Other questionnaires used in classification and score means for the total sample as well as each appropriateness subgroup appear in the supplementary material. The focus of this report is on the key outcome measures.

**Table 1:** Baseline characteristics for patients with missing data versus those without missing data at 6 months

	Missing data *N* = 52	Complete data *N* = 282	*p*-value
	Mean (SD)	Mean (SD)	
**Gender** (female), *n* (%)	35 (67.3%)	184 (65.2%)	0.87
**Age**	71.0 (6.8)	70.9 (8.3)	0.92
**Appropriateness classification**, *n* (%)			
Appropriate	31 (59.6%)	142 (50.4%)	
Inappropriate	9 (17.3%)	50 (17.7%)	0.40
Uncertain	12 (23.1%)	90 (31.9%)	
**WOMAC** (range 0–100) (best to worst)			
Pain	52.02 (19.30)	53.61 (19.17)	0.58
Stiffness	53.85 (25.43)	53.01 (25.57)	0.83
Function	57.87 (19.20)	57.59 (19.33)	0.92
**SF12v1** (range 0–100) (worst to best)			
PCS	26.71 (7.55)	28.17 (7.00)	0.19
MCS	46.00 (15.02)	45.04 (14.80)	0.68
**KSS**			
Satisfaction with: (range 0–8) (lowest to highest)			
Pain level while sitting^a^	3.76 (2.0)	3.44 (2.1)	0.78
Pain level while lying in bed^a^	4.08 (2.1)	3.60 (2.1)	0.26
Function while getting out of bed^a^	2.49 (2.1)	2.17 (1.7)	0.90
Function performing household duties^a^	2.40 (1.7)	2.09 (1.5)	0.18
Function performing leisure activities^a^	1.76 (1.7)	1.63 (1.6)	0.67
Total satisfaction score (range 0–40)	14.4 (7.7)	12.9 (6.8)	0.18

^a^*U*-Mann-Whitney

*SD* Standard deviation; *WOMAC* Western Ontario and McMaster Universities Osteoarthritis Index; *SF-12* Short-Form-12 Health Survey; *PCS* Physical Component Summary; *MCS* Mental Component Summary; *KSS* Knee Society Clinical Rating

### Baseline score comparisons

At baseline, there were significant differences amongst appropriateness groups in all WOMAC, SF-12, and KSS satisfaction scores (Table 2). Regarding the WOMAC questionnaire, gradient differences amongst the three groups were found, with worst to best from Appropriate to Uncertain to Inappropriate, respectively, in the three domains (*p* < 0.001). These significant gradient differences also were found for each of the five satisfaction questions and the overall satisfaction score of the KSS (*p* < 0.001).

**Table 2:** Baseline self-report measures by appropriateness groups

	Appropriate^a^ *N* = 142	Uncertain^b^ *N* = 90	Inappropriate^c^ *N* = 50	*p*-value
	Mean (SD)	Mean (SD)	Mean (SD)	
**WOMAC** (range 0–100) (best to worst)				
Pain	65.8 (13.9)^b,c^	47.6 (13.1)^a,c^	29.8 (12.6)^a,b^	< 0.001
Stiffness	63.6 (24.4)^b,c^	47.4 (20.1)^a,c^	33.2 (22.8)^a,b^	< 0.001
Function	69.7 (14.1)^b,c^	50.9 (13.3)^a,c^	35.3 (15.2)^a,b^	< 0.001
**SF12v1** (range 0–100) (worst to best)				
PCS	26.3 (6.1)^c^	28.3 (6.2)^c^	33.0 (8.5)^a,b^	< 0.001
MCS	41.7 (14.9)^b,c^	48.0 (14.1)^a^	48.5 (13.9)^a^	0.002
**KSS**				
Satisfaction with: (range 0–8) (lowest to highest)				
Pain level while sitting^*^	2.6 (1.7)^b,c^	4.0 (2.1)^a,c^	4.7 (2.1)^a,b^	< 0.001
Pain level while lying in bed^*^	2.9 (1.9)^b,c^	4.0 (2.0)^a,c^	5.0 (2.1)^a,b^	< 0.001
Function while getting out of bed^*^	1.6 (1.4)^b,c^	2.2 (1.5)^a,c^	3.5 (2.0)^a,b^	< 0.001
Function performing household duties^*^	1.5 (1.2)^b,c^	2.2 (1.4)^a,c^	3.3 (1.8)^a,b^	< 0.001
Function performing leisure activities^*^	1.2 (1.4)^c^	1.7 (1.5)^c^	2.6 (2.0)^a,b^	< 0.001
Total satisfaction score (range 0–40)	9.9 (5.2)^b,c^	14.1 (6.2)^a,c^	18.8 (7.1)^a,b^	< 0.001

^*^Kruskal-Wallis

Superscript letters indicate differences among the appropriateness categories (^a^appropriate, ^b^uncertain, ^c^inappropriate) by means of the Scheffé test for multiple comparisons

*SD* Standard deviation; *WOMAC* Western Ontario and McMaster Universities Osteoarthritis Index; *SF-12* Short-Form-12 Health Survey; *PCS* Physical Component Summary; *MCS* Mental Component Summary; *KSS* Knee Society Clinical Rating

### Comparisons of 6-month journey (change) scores

There were differences amongst the three groups (*p* < 0.001) in WOMAC domains (Table 3). The KSS satisfaction scores demonstrated differences between Appropriate and Inappropriate groups (*p* < 0.001), with a gradient in improvement from Appropriate to Uncertain to Inappropriate. In all cases, the patients classified as Appropriate had a greater improvement than Uncertain and for Uncertain compared with Inappropriate. No differences amongst groups were found for the PCS, but there were differences in the MCS change scores between Appropriate and Inappropriate groups (*p* = 0.03).

**Table 3:** Changes (journey) and final scores (destination) in self-report measures at 6 months by appropriateness groups

	Changes (journey)				Final scores at 6 months, destination			
	Appropriate^a^ *N* = 142	Uncertain^b^ *N* = 90	Inappropriate^c^ *N* = 50	*p*-value	Appropriate *N* = 142	Uncertain *N* = 90	Inappropriate *N* = 50	*p*-value
	Mean (SD)	Mean (SD)	Mean (SD)		Mean (SD)	Mean (SD)	Mean (SD)	
**WOMAC** (range 0–100) (best to worst)								
Pain	43.2 (21.6)^b,c^	26.4 (18.1)^a,c^	12.8 (17.8)^a,b^	< 0.001	22.7 (17.1)	21.2 (16.5)	17.0 (15.1)	0.11
Stiffness	36.6 (32.5)^b,c^	24.7 (25.3)^a,b^	11.5 (25.9)^a,b^	< 0.001	26.9 (20.4)	22.6 (19.7)	21.7 (15.5)	0.14
Function	42.3 (22.5)^b,c^	26.8 (17.7)^a,c^	13.7 (18.5)^a,b^	< 0.001	27.3 (18.6)	24.1 (17.7)	21.5 (15.6)	0.11
**SF12v1** (range 0–100) (worst to best)								
PCS	14.0 (12.4)	13.3 (11.9)	9.3 (11.5)	0.10	40.0 (11.0)	41.6 (11.2)	42.5 (10.8)	0.33
MCS	6.1 (14.9)^c^	2.0 (14.6)	−0.1 (13.5)^a^	0.03	48.1 (11.6)	49.9 (9.8)	46.5 (13.1)	0.25
**KSS**								
Satisfaction with: (range 0–8) (lowest to highest)								
Pain level while sitting^*^	3.5 (2.5)^b,c^	2.0 (2.5)^a^	1.6 (2.6)^a^	< 0.001	6.1 (1.9)	6.0 (1.9)	6.3 (1.8)	0.74
Pain level while lying in bed^*^	3.3 (2.5)^b,c^	2.2 (2.6)^a^	1.5 (2.7)^a^	< 0.001	6.2 (2.1)	6.1 (2.0)	6.4 (1.6)	0.58
Function while getting out of bed^*^	4.2 (2.4)^c^	3.4 (2.4)	2.4 (2.7)^a^	< 0.001	5.8 ()2.0	5.7 (2.0)	5.9 (1.7)	0.80
Function performing household duties^*^	3.9 (2.4)^c^	3.4 (2.1)	2.4 (2.6)^a^	0.001	5.5 (2.0)	5.6 (1.9)	5.8 (1.9)	0.74
Function performing leisure activities^*^	4.1 (2.2)^c^	3.7 (2.1)^c^	2.6 (2.6)^a,b^	0.001	5.3 (2.1)	5.4 (2.1)	5.3 (2.1)	0.88
Total satisfaction score (range 0–40)	19.0 (10.2)^b,c^	14.8 (9.5)^a^	10.4 (10.9)^a^	< 0.001	28.9 (9.3)	28.7 (9.2)	29.4 (8.1)	0.91

^*^Kruskal-Wallis

Superscript letters indicate statistically significant differences among the three appropriateness categories (^a^appropriate, ^b^uncertain, ^c^inappropriate) by means of the Scheffé test for multiple comparisons

*SD* Standard deviation; *WOMAC* Western Ontario and McMaster Universities Osteoarthritis Index; *SF-12* Short-Form-12 Health Survey; *PCS* Physical Component Summary; *MCS* Mental Component Summary; *KSS* Knee Society Clinical Rating

Results regarding patients attaining the baseline adjusted WOMAC pain MCID (Table 4) indicated that 66.4% for the Appropriate group, 54.4% for Uncertain group, and 48.0% for Inappropriate (*p* = 0.04) attained the MCID. Results were similar for WOMAC function MCID (*p* = 0.004).

**Table 4:** Percentage of patients attaining the WOMAC Pain and Function MCID adjusted for baseline quartile

		Sample size (***n***) and patients attaining MCID						
Baseline quartiles (range)	MCID		Appropriate		Uncertain		Inappropriate	***p***-value
		n^*^	Change ≥ MCID *n* (%)	n^*^	Change ≥ MCID *n* (%)	n^*^	Change ≥ MCID *n* (%)	
Pain								
Q1 (least pain) (≤ 40)	12.4	6	5 (83.3)	34	20 (58.8)	43	22 (51.1)	
Q2 (40.1–50)	25.1	13	9 (64.3)	41	20 (48.8)	6	2 (33.3)	
Q3 (50.1–65)	32.2	60	37 (61.7)	7	6 (85.7)	0	–	
Q4 (worst pain) (> 65)	47.9	61	42 (68.9)	8	3 (37.5)	1	0 (0)	
Total attaining MCID by quartile			93 (66.4)		49 (54.4)		24 (48.0)	0.04
Function								
Q1 (least function) (< 45)	15.9	5	2 (40.0)	29	20 (68.9)	38	13 (34.2)	
Q2 (45–56)	27.6	21	11 (52.4)	41	22 (53.7)	8	3 (37.5)	
Q3 (56.1–72.5)	37.2	55	30 (54.5)	12	5 (41.7)	3	1 (33.3)	
Q4 (worst function) (> 72.5)	47.5	60	41 (68.3)	8	3 (37.5)	1	0 (0)	
Total attaining MCID by quartile			84 (59.6)		50 (55.5)		17 (34.0)	0.004

^*^Sample size in each quartile by appropriateness category

*MCID* Minimal clinically important difference; *Q* Quartile; *WOMAC* Western Ontario and McMaster Universities Osteoarthritis Index

### Comparisons of 6-month destination (final outcome) scores

None of the scales showed statistically significant differences among the three appropriateness groups at 6 months (Table 3). Applying the OMERACT-OARSI criteria to the total sample, the percentage of responders was 90.8% for the Appropriate group, 88.9% for Uncertain, and 64.0% for the Inappropriate group (*p* < 0.001).

### Satisfaction: baseline and destination comparisons

Dichotomized patient ratings of satisfaction appear in Table 5. At baseline, there were statistically significant differences (*p* < 0.001) in the five questions. At baseline, a higher percentage of satisfied patients were classified as Inappropriate as compared to appropriate and uncertain subgroups, with percentages ranging from 13.3 to 22.4% satisfied with function items and approximately 45% satisfied for pain items. At 6 months, there were no differences in the percentages of satisfied patients amongst appropriateness groups.

**Table 5:** Individual satisfaction questions stratified by either pain or function and whether scores were obtained at baseline or 6 months following TKA

	Satisfaction with pain^a^				Satisfaction with function^a^					
	While sitting		While lying in bed		While getting out of bed		Performing household duties		Performing leisure activities	
	Baseline *n* (%)	6 months *n* (%)	Baseline *n* (%)	6 months *n* (%)	Baseline *n* (%)	6 months *n* (%)	Baseline *n* (%)	6 months *n* (%)	Baseline *n* (%)	6 months *n* (%)
Appropriateness										
Appropriate	12 (8.8)	105 (77.2)	21 (15.4)	104 (76.5)	3 (2.2)	98 (72.0)	2 (1.5)	88 (64.7)	2 (1.5)	72 (55.4)
Uncertain	35 (38.9)	69 (76.7)	26 (29.2)	68 (76.4)	4 (4.5)	59 (66.3)	3 (3.3)	59 (65.5)	3 (3.6)	48 (57.1)
Inappropriate	22 (44.9)	39 (79.6)	24 (49.0)	41 (83.7)	11 (22.4)	35 (71.4)	10 (20.4)	34 (69.4)	6 (13.3)	26 (57.8)
*p*-value	< 0.001	0.9	< 0.001	0.5	< 0.001	0.7	< 0.001	0.8	0.001	0.5

^a^Scores are reported separately for the two pain items and the three function items. All satisfaction scores were dichotomized to indicate whether the patient was either satisfied (i.e., very satisfied or satisfied) or unsatisfied (neutral, dissatisfied, or very dissatisfied). The table reports the sample size (*n*) and percentage of patients for each appropriateness classification and for baseline and 6-month time points

*TKA* Total knee arthroplasty

## Discussion

The main objective of our study was to prospectively test the validity of our RAND/UCLA appropriateness system by recruiting a sample of patients undergoing TKA. Our first hypothesis was confirmed. Baseline scores for WOMAC Pain and Function as well as satisfaction with current status were statistically different amongst the three appropriateness groups. The most severely affected patients were classified as Appropriate and the least severely affected patients were classified as Inappropriate. In addition to statistical differences, their magnitude of differences was quite large, for example, > 30 points between Appropriate and Inappropriate groups in baseline pain and function.

At baseline, the differences in the percentage of patients satisfied or very satisfied with their current pain level or knee function were even higher, between Inappropriate and Appropriate groups. Differences in HRQoL, measured by SF-12, were consistent with other baseline measures, although their magnitudes were less, possibly because generic HRQoL measures are not designed to optimize between patient differences. Patients classified as Appropriate had worse HRQoL relative to Uncertain or Inappropriate groups.

Data comparing Appropriate to Uncertain groups differ from a modified version of our first-generation TKA appropriateness tool where baseline scores for the Uncertain and Appropriate groups were very similar [18]. However, the baseline results of the current study are more in line with a prior study conducted in Spain [26], where we found the Appropriate group had more severe pain and functional deficits relative to the Uncertain group which, in turn, was worse than the Inappropriate group. Our suspicion is that the additional criteria added to our system (i.e., psychological factors, pain catastrophizing, and comorbidities) combined with deletion of older criteria (e.g., range of motion and knee stability) resulted in a clearer separation of Appropriate, Uncertain, and Inappropriate classifications as compared to the original (first generation) [5] or modified [18] system. We see this as a strength of the system, given that in theory, there should be clear separation and measurable baseline differences among the three appropriateness categories.

Our second hypothesis was also supported. Greater improvements occurred in patients classified as Appropriate relative to patients in the Inappropriate group for WOMAC scores and satisfaction. The percentage of patients attaining their baseline adjusted MCID also support this hypothesis. The Uncertain group demonstrated a pattern of improvement that mirrored the baseline findings. That is, the Uncertain group had improvements in WOMAC scores that fell approximately midway between the Appropriate and Inappropriate groups. Similar results were obtained when considering OMERACT-OARSI responder criteria, a finding also reported in prior work [27], but again there are differences amongst groups, with the Appropriate group demonstrating a higher percentage of patients considered as responders.

Finally, our hypothesis about final scores was supported. We did not find significant differences in 6-month WOMAC scores among the three classification groups. Much like our prior outcome studies [18, 26], the three appropriateness groups all ended up with similar final pain and function scores. A novel and important finding in the current study is that final satisfaction scores among the three appropriateness groups also were not statistically different. We do not believe that a ceiling effect explains the lack of difference among the three groups at 6 months. All groups had additional room for improvement across all outcome measures.

When considering our results in total, our second-generation appropriateness system performed reasonably well in that the baseline differences among the three groups were more substantial than prior studies [18, 26], creating a clearer distinction among the three groups. However, 6-month changes and final destination outcome findings were more nuanced. While change scores were smaller for the Inappropriate and Uncertain groups relative to the Appropriate group, all groups ended up in approximately the same place and satisfaction at 6 months was not different among the three groups. These data suggest that patients classified as Inappropriate derived less benefit (i.e., their change scores were smaller) but they were, as a group, as satisfied as the other two appropriateness groups. This begs the question of whether TKA decision-making should be driven by the magnitude of benefit, that is, the change score from baseline, or whether it should be driven by patient satisfaction and pain and function at the final outcome time point (i.e., final outcome was assessed 6 months after surgery), or some combination of both. Our study cannot answer this question but will hopefully provide a stimulus for developing consensus on this issue. Losina and Katz posed a similar question [19]. Our study further informs deliberations on the question of appropriateness in that we found, for the first time, that satisfaction is equally high among the three appropriateness groups.

Another important finding is that the Uncertain group represented 31.9% of the sample. Baseline scores were different in nearly all dimensions for the Uncertain group compared to the other two groups and with intermediate values. It is possible that additional indication criteria variables would help to clarify whether this Uncertain group should actually be classified as Appropriate or Inappropriate. Additional work is needed to determine the reasons for this relatively large proportion of patients classified as Uncertain. An alternative explanation, given that TKA is an elective procedure, is that even with the application of a new classification system with multiple criteria for judging appropriateness, substantial uncertainty persists regarding determinations of appropriateness for surgery.

There are limitations to our study. It is likely that the use of the Hospital Anxiety and Depression Scale, and Pain Catastrophizing Scale is not likely to be common in daily practice and their use by surgeons may not have been optimal. While we relied on current evidence to drive the selection of these measures and their cutpoints, it may be that surgeons are unfamiliar with these measures and the new prognostic evidence that support their use [3, 28]. We believe that since they are variables supported by evidence to indicate the likely prognosis of TKA, their measurement should be incorporated, much like pain or functional capacity or radiographic OA, in a standardized way. Finally, our power analysis indicated we needed 185 patients in the “appropriate” category and we ended up with 142. However, we still found significant differences among the classifications for percentage of patients meeting or exceeding the MCID for WOMAC scores (see Table 4).

Our data were collected in Spain and there is uncertainty regarding the extent to which these findings generalize to other countries. Finally, while our validity analyses showed fairly dramatic differences among the three classification groups across baseline and change scores, there were no significant final outcome differences. Before one can conclude that this system may not demonstrate clear and strong differences among appropriate versus inappropriate and inconclusive groups at the final outcome, additional cohorts of patients should be studied to see if the more nuanced findings reported here are consistent across different samples.

Our outcomes were measured 6 months following surgery, and it may be the results could vary with longer follow-up. However, data suggest that changes from 6 to 12 months are minimal for the WOMAC and likely other pain and function self-reported outcomes [29]. Finally, our study included patients who already consented to undergo TKA, so we do not know if variables considered as important prior to obtaining patient consent for TKA such as the extent of social support, prior treatment, or expectations had been properly managed.

Future work on appropriateness criteria should be focused on external validation of our proposed system on patients from different countries and different healthcare systems. External validation will be important in judging the extent to which our system might impact TKA decision-making in other countries. Additionally, further development of our system should be encouraged as evidence identifies additional important indicators of outcome.

## Conclusion

Our results generally supported the validity of our TKA appropriateness classification system though the clinical impact of these findings is likely to be modest. The findings highlight a critical issue in TKA decision-making going forward. Whether appropriateness should be driven primarily by the magnitude of improvement over time or by patient satisfaction and pain and functional status following recovery is unknown. Consensus development on this issue should be a high priority for stakeholders involved with TKA healthcare delivery.

## Supplementary Information

**Additional file 1.** Methods for developing the RAND/UCLA based appropriateness classification system.

## Data Availability

The data that support the findings of this study are potentially available from Dr. Bilbao, but restrictions apply to the availability of these data and restrictions by the funding agency.

## Abbreviations

TKA: Total knee arthroplasty
OA: Osteoarthritis
AAOS: American academy of orthopaedic surgeons
RUAM: RAND/UCLA appropriateness method
HRQoL: Health-related quality of life
WOMAC: Western Ontario and McMaster universities osteoarthritis index
SF-12: Short-form-12
MCS: Mental component summary
PCS: Physical component summary
KSS: Knee society clinical rating system
ANOVA: Analysis of variance
MCID: Minimal clinically important difference
OMERACT-OARSI: Outcome measures in rheumatology-osteoarthritis research society

## References

[1] Sloan M, Premkumar A, Sheth NP (2018). Projected volume of primary total joint arthroplasty in the U.S., 2014 to 2030. J Bone Jt Surg Am Vol.

[2] Fraenkel L, Suter L, Weis L, Hawker GA (2014). Variability in recommendations for total knee arthroplasty among rheumatologists and orthopedic surgeons. J Rheumatol.

[3] Dumenci L, Perera R, Keefe F, Ang D, Slover J, Jensen M (2019). Model-based pain and function outcome trajectory types for patients undergoing knee arthroplasty: a secondary analysis from a randomized clinical trial. Osteoarthritis Cartilage.

[4] Naylor CD, Williams JI (1996). Primary hip and knee replacement surgery: Ontario criteria for case selection and surgical priority. Qual Health Care.

[5] Escobar A, Quintana JM, Arostegui I, Azkarate J, Guenaga JI, Arenaza JC (2003). Development of explicit criteria for total knee replacement. Int J Technol Assess Health Care.

[6] Frankel L, Sanmartin C, Hawker G, De CC, Dunbar M, Bohm E (2016). Perspectives of orthopaedic surgeons on patients’ appropriateness for total joint arthroplasty: a qualitative study. J Eval Clin Pract.

[7] Verra WC, Witteveen KQ, Maier AB, Gademan MGJ, van der Linden HMJ, Nelissen RGHH (2016). The reason why orthopaedic surgeons perform total knee replacement: results of a randomised study using case vignettes. Knee Surg Sport Traumatol Arthrosc.

[8] Conner-Spady BL, Marshall DA, Hawker GA, Bohm E, Dunbar MJ, Frank C (2014). You’ll know when you’re ready: a qualitative study exploring how patients decide when the time is right for joint replacement surgery. BMC Health Serv Res.

[9] Schmitt J, Lange T, Günther K-P, Kopkow C, Rataj E, Apfelbacher C, Aringer M, Böhle E, Bork H, Dreinhöfer K, Friederich N, Frosch KH, Gravius S, Gromnica-Ihle E, Heller KD, Kirschner S, Kladny B, Kohlhof H, Kremer M, Leuchten N, Lippmann M, Malzahn J, Meyer H, Sabatowski R, Scharf HP, Stoeve J, Wagner R, Lützner J (2017). Indication criteria for total knee arthroplasty in patients with osteoarthritis – a multi-perspective consensus study. Z Orthop Unfall.

[10] Quintana JM, Arostegui I, Escobar A, Azkarate J, Goenaga JI, Lafuente I (2008). Prevalence of knee and hip osteoarthritis and the appropriateness of joint replacement in an older population. Arch Intern Med.

[11] Riddle DL, Jiranek WA, Hayes CW (2014). Use of a validated algorithm to judge the appropriateness of total knee arthroplasty in the United States: a multicenter longitudinal cohort study. Arthritis Rheumatol.

[12] Ghomrawi HMK, Mushlin AI, Kang R, Banerjee S, Singh JA, Sharma L et al. Examining timeliness of total knee replacement among patients with knee osteoarthritis in the U.S. J Bone Jt Surg Am Vol. 2020;102(6):468–76. 10.2106/JBJS.19.00432. 10.2106/JBJS.19.00432 PMC7508265 31934894

[13] Skou ST, Roos EM, Laursen MB, Rathleff MS, Arendt-Nielsen L, Simonsen O, Rasmussen S (2015). A randomized, controlled trial of total knee replacement. N Engl J Med.

[14] Fitch K, Bernstein SJ, Aguilar MD, Burnand B, LaCalle JR, Lazaro P, et al. The RAND/UCLA appropriateness method user’s manual. April 2001. http://www.rand.org/pubs/monograph_reports/MR1269.html. Accessed June 2020.

[15] Lawson EH, Gibbons MM, Ko CY, Shekelle PG (2012). The appropriateness method has acceptable reliability and validity for assessing overuse and underuse of surgical procedures. J Clin Epidemiol.

[16] Shekelle PG, Park RE, Kahan JP, Leape LL, Kamberg CJ, Bernstein SJ (2001). Sensitivity and specificity of the RAND/UCLA Appropriateness Method to identify the overuse and underuse of coronary revascularization and hysterectomy. J Clin Epidemiol.

[17] American Academy of Orthopaedic Surgeons (2016). Appropriate use criteria for the surgical management of osteoarthritis of the knee.

[18] Riddle DL, Perera RA, Jiranek WA, Dumenci L (2015). Using surgical appropriateness criteria to examine outcomes of total knee arthroplasty in a United States sample. Arthritis Care Res.

[19] Losina E, Katz JN (2012). Total knee replacement: pursuit of the paramount result. Rheumatology(Oxford).

[20] Escobar-Martinez A, Perera RA, Riddle DL. Development and underlying structure of a second-generation appropriateness classification system for total knee arthroplasty. Arthritis Care Res (Hoboken). 2020; 10.1002/acr.24169. Online ahead of print. 10.1002/acr.24169 32100952

[21] Escobar A, Quintana J, Bilbao A, Azkárate J, Güenaga JI (2002). Validation of the Spanish version of the WOMAC questionnaire for patients with hip or knee osteoarthritis. Western Ontario and McMaster Universities Osteoarthritis Index. Clin Rheumatol.

[22] Gandek B, Ware JE (1998). Aaronson et al. NK. Cross-validation of item selection and scoring for the SF-12 Health Survey in nine countries: results from the IQOLA Project. International Quality of Life Assessment. J Clin Epidemiol.

[23] Ares O, Castellet E, Maculé F, León V, Montañez E, Freire A, Hinarejos P, Montserrat F, Amillo JR (2013). Translation and validation of “The Knee Society Clinical Rating System” into Spanish. Knee Surg Sport Traumatol Arthrosc.

[24] Escobar A, Riddle DL (2014). Concordance between important change and acceptable symptom state following knee arthroplasty: the role of baseline scores. Osteoarthritis Cartilage.

[25] Pham T, van der HD ARD, Anderson JJ, Bellamy N, Hochberg M (2004). OMERACT-OARSI initiative: Osteoarthritis Research Society International set of responder criteria for osteoarthritis clinical trials revisited. Osteoarthritis Cartilage.

[26] Quintana JM, Escobar A, Arostegui I, Bilbao A, Azkarate J, Goenaga JI, Arenaza JC (2006). Health-related quality of life and appropriateness of knee or hip joint replacement. Arch Intern Med.

[27] Escobar A, Gonzalez M, Quintana JM, Vrotsou K, Bilbao A, Herrera-Espineira C (2012). Patient acceptable symptom state and OMERACT-OARSI set of responder criteria in joint replacement. Identification of cut-off values. Osteoarthritis Cartilage.

[28] Sullivan M, Tanzer M, Stanish W, Fallaha M, Keefe FJ, Simmonds M, Dunbar M (2009). Psychological determinants of problematic outcomes following total knee arthroplasty. Pain.

[29] Canfield M, Savoy L, Cote MP, Halawi MJ (2020). Patient-reported outcome measures in total joint arthroplasty: defining the optimal collection window. Arthroplasty Today.

