# Supplementary material for: Validation of a second-generation appropriateness classification system for total knee arthroplasty: a prospective cohort study
Source: J Orthop Surg Res. 2021 Mar 29;16:227. doi: 10.1186/s13018-021-02371-z (PMC8006353; doi:10.1186/s13018-021-02371-z)
Supplement: Supplementary file 1 — Additional file 1. Methods for developing the RAND/UCLA based appropriateness classification system. [file 13018_2021_2371_MOESM1_ESM.docx]

**ADDITIONAL FILE 1**

**Methods for developing the RAND/UCLA based appropriateness classification system**

We followed recommendations originally developed by Brook [1] and fully described in the RAND/UCLA User’s Manual [2] to develop the second-generation appropriateness criteria for total knee arthroplasty (TKA). The process has the following steps.

**Assumptions for the expert panels**

Prior to initiating of the RAND/UCLA process, the expert panels were instructed to assume the following: 1) only primary TKA for osteoarthritis (OA) would be considered, 2) if hip OA was also present, it would be appropriately managed, 3) prior surgical treatment and prior and current medications for knee OA were appropriately managed, 4) active infection, quadriceps tendon rupture or severe peripheral vascular disease had effectively been ruled out, 5) expectations regarding outcome following TKA was appropriately addressed, 6) social support (e.g., caregiver or spouse/partner) for the TKA recipient would be available, and 7) body mass index was less than 40kg/m^2^.

**Identification and review of the literature**

The first step was to conduct a comprehensive review of the scientific literature to gather available evidence about the use, efficacy, effectiveness, opinions, outcomes and risks of TKA. A specially trained reviewer synthesized the available scientific evidence about TKA from inception to May, 2016 in The Cochrane Library, EMBASE (OVID) and PsycINFO (OVID) databases.

**Expert panel #1. Research Team**

The purpose of the literature review was to provide members of expert panel #1 with an up-to-date source of scientific content to identify key indication criteria that would serve as the basis for writing the scenarios. Expert Panel #1 included six orthopaedic surgeons, one psychologist, one statistician, one social worker and one clinical epidemiologist.

There were a total of eight indication criteria identified by expert panel #1 based on the literature review (see Supplementary Table 1). Of these criteria, four had dichotomous responses, three had trichotomous responses and one had four responses. The definitions for the response cutpoints for the eight indication criteria were developed by expert panel #1 prior to writing the scenarios. For the pain and functional limitation criteria, the Spanish validated Western Ontario and McMaster Universities Osteoarthritis Index (WOMAC) Pain and WOMAC Function scales [3] respectively were chosen by expert panel #1. The WOMAC scales each ranged from 0 to 100 with higher scores equating to worse pain or function. Each had three categories (i.e., slight with scores of <35, moderate with scores of 35 to 50 for Pain and 35 to 54 for Function, and severe with score >50 for Pain and > 54 for Function). For “psychological factors” the panel chose to use the recommended cutpoint of 10 to differentiate among those without versus with clinical anxiety and depression as measured by the Spanish validated version of the Hospital Anxiety and Depression Scale [4] and for pain catastrophizing the panel chose a cutpoint of 30 to dichotomize low versus high catastrophizing based on the Spanish validated Pain Catastrophizing Scale [6]. For comorbidity, the panel chose a cutpoint of 0 to differentiate among persons without from those with one or more comorbidity (yes) (see Supplementary Table 1). For the Radiology criterion, a Kellgren and Lawrence grade cutpoint of 2 was used. Knee OA localization was dichotomized to either unicompartmental or multicompartmental disease.

Expert panel #1 used the eight criteria to write a series of brief clinical vignettes incorporating the eight criteria. The scenarios had to be mutually exclusive and exhaustive as well as clinically feasible for persons considering TKA. The intention was to cover virtually all clinical patterns of knee problems for which TKR might be considered. Combining these variables in a factorial design would result in n = 1,152 scenarios (2^5^ x 3^2^ x 4^1^). The expert panel concluded that for scenarios with age > 85 years, the knee osteoarthritis location (i.e., number of compartments affected) did not influence classification and therefore 1,008 scenarios were written.

**Expert panel #2**

A national panel of clinical experts from Spain were selected. The research team asked the Spanish Knee Society to nominate nationally recognized specialists in TKA care. After the initial contact, twelve specialists, ten orthopaedic surgeons and two rheumatologists agreed to participate. Panelists were provided with the literature review, the list of indication criteria and the 1,008 scenarios. Expert panel #2 was asked to rate each scenario for the appropriateness of TKA, considering the average patient and average physician in 2016.

Appropriate was defined as meaning that the expected health benefit of TKA exceeds the expected negative consequences by a sufficiently wide margin to make TKA worth performing [2]. Inappropriate was defined as the risk of negative consequences outweighed expected benefits and Uncertain was defined as the situation in which benefit and risk could not be estimated. The indication criteria and scenarios were reviewed and approved by expert panel #2 prior to scenario rating.

**Scenario ratings by expert panel #2**

Ratings took place over two rounds using a modified Delphi method . The first round was performed at the individual level and the second round during a one-day in-person panel meeting in which each panelist received the results of his/her own scores for each scenario, and the anonymized ratings made by the other members. After discussion of scenarios with disagreement during round one (n = 27), the panelists were able to revise their ratings. The aim was not to reach consensus but to identify the level of agreement among the participants after discussion.

Ratings were scored on a nine-point scale. The medians for each scenario could lie within the ranges of 1 to 3 for a rating of Inappropriate, 4 to 6 for a rating of Uncertain and 7 to 9 for Appropriate. Use of TKA was considered Appropriate if the panel's median rating was between 7-9 with agreement (defined below), Uncertain if the panel’s median rating was between 4-6 with agreement or any median score with disagreement (defined below). A rating of Inappropriate was given if the median rating was between 1-3 with agreement. Agreement was established when one third or less of the panelists’ scores fell outside the median categorical appropriateness rating. Disagreement occurred when greater than one third of panelists disagreed on the median categorical rating. Scenarios were rated as indeterminate when criteria for either agreement or disagreement were not met.

**Supplementary Table 1. Indication Criteria and Appropriateness Ratings of 1,008 scenario**

| Indication Criteria. Measurement Scale | Scenario Sample Size (%) |
| --- | --- |
| AGE |  |
| < 55 years | 288 (28.6) |
| 55-65 years | 288 (28.6) |
| > 66-85 years | 288 (28.6) |
| > 85 years | 144 (14.3) |
| RADIOLOGY (Kellgren and Lawrence (KL) grade) |  |
| KL ≤ 2 | 504 (50.0) |
| KL ≥ 3 | 504 (50.0) |
| KNEE OSTEOARTHRITIS LOCALIZATION |  |
| Unicompartmental | 432 (42.9) |
| More than one compartment | 432 (42.9) |
| Either uni or multiple compartment^*^ | 144 (14.3) |
| PAIN (Measured with WOMAC Pain Scale scored 0 (best) to 100 (worst)) |  |
| Slight (< 35) | 336 (33.3) |
| Moderate (35-50) | 336 (33.3) |
| Severe (> 50) | 336 (33.3) |
| FUNCTION (Measured with WOMAC Function Scale scored 0 (best) to 100 (worst)) |  |
| Slight (< 35) | 336 (33.3) |
| Moderate (35-54) | 336 (33.3) |
| Severe (> 54) | 336 (33.3) |
| PSYCHOLOGICAL FACTORS (Anxiety or depression measured with Hospital Anxiety and Depression Scale (HADS)) |  |
| ≤ 10 in both anxiety and depression | 504 (50.0) |
| > 10 on either anxiety or depression | 504 (50.0) |
| PAIN CATASTROPHIZING (Measured with Pain Catastrophizing Scale) |  |
| ≤ 30 | 504 (50.0) |
| >30 | 504 (50.0) |
| COMORBIDITIES |  |
| No | 504 (50.0) |
| At least one^+^ | 504 (50.0) |
| Appropriateness Rating |  |
| Inappropriate | 671 (66.6) |
| Uncertain | 256 (25.4) |
| Appropriate | 81 (8.0) |

^*^ For scenarios with age > 85 years, the expert panel indicated that unicompartmental or multicompartmental disease would be treated in the same way.

^+^ Relevant comorbidities are the following: osteoporosis, asthma, chronic obstructive pulmonary disease, heart failure, Parkinson’s disease, multiple sclerosis, stroke, chronic back pain.

**Questionnaires**

The Hospital Anxiety and Depression Scale (HADS) consist of 14 items from which two subscales are derived: anxiety and depression scales. Each subscale ranges from 0 to 21. Recommended cut-off points are >10 as probable psychological distress. The HADS has been validated in Spanish [4]. The mean scores for the HADS Anxiety scale for the sample of 282 patients was 7.92 (SD = 5.07) and for the HADS Depression scale the mean score was 6.96 (SD = 4.30). The mean HADS Anxiety score for the Appropriate subgroup was 9.1 (SD = 5.0), 7.1 (SD = 4.8) for the Uncertain subgroup and 6.1 (SD = 5.0) for the Inappropriate subgroup. The mean HADS Depression score for the Appropriate subgroup was 7.8 (SD = 4.3), 6.7 (SD = 4.4) for the Uncertain subgroup and 5.2 (SD = 3.6) for the Inappropriate subgroup.

The Pain Catastrophizing Scale (PCatS) is a 13 items questionnaire conforming a total scale ranging from 0 (no catastrophizing) to 52 (severe catastrophizing). It has been validated into Spanish [6]. We dichotomized the scores such that vignettes were considered to have pain catastrophizing with scores of 30 or greater and score of less than 30 were considered to be negative on pain catastrophizing. The mean score for the PCatS for the sample of 282 patients was 22.85 (SD = 14.96). The mean PCatS for the Appropriate subgroup was 27.2 (SD =14.2), 21.0 (SD = 14.4) for the Uncertain subgroup and 14.3 (SD = 13.8) for the Inappropriate subgroup.

Comorbidity status was determined by asking patients to indicate which of the following diagnoses they had been given: Osteoporosis, Asthma, Chronic Obstructive Pulmonary Disease, Heart Failure, Neurological diseases (Parkinson, Multiple Sclerosis, Stroke), Back pain. A total count for each patient was recorded as either 0 or at least 1 comorbidity. A total of 57% of the Appropriate subgroup scored a 0, 46.7% of the Uncertain subgroup scored a 0 and 64% of the Inappropriate subgroup scored a 0.

**REFERENCES**

[1] Brook RH, Chassin MR, Fink A, Solomon DH, Kosecoff J, Park RE. A method for the detailed assessment of the appropriateness of medical technologies. Int J Technol Assess Health Care 1986;2:53–63. doi:10.1017/S0266462300002774.

[2] Fitch K, Bernstein SJ, Aguilar MD, Burnand B, LaCalle JR, Lazaro P, et al. The RAND/UCLA Appriateness Method User’s Manual 2001. http://www.rand.org/pubs/monograph_reports/MR1269.html. Accessed April, 2018.

[3] Escobar A, Quintana J, Bilbao A, Azkárate J, Güenaga JI. Validation of the Spanish version of the WOMAC questionnaire for patients with hip or knee osteoarthritis. Western Ontario and McMaster Universities Osteoarthritis Index. Clin Rheumatol 2002;21:466–71. doi:10.1007/s100670200117.

[4] Herrero MJ, Blanch J, Peri JM, De Pablo J, Pintor L, Bulbena A. A validation study of the hospital anxiety and depression scale (HADS) in a Spanish population. Gen Hosp Psychiatry 2003;25:277–83. doi:10.1016/S0163-8343(03)00043-4.

[5] Zigmond AS, Snaith RP. The Hospital Anxiety and Depression Scale. Acta Psychiatr Scand 1983;67:361–70. doi:10.1111/j.1600-0447.1983.tb09716.x.

[6] García Campayo J, Rodero B, Alda M, Sobradiel N, Montero J, Moreno S. [Validation of the Spanish version of the Pain Catastrophizing Scale in fibromyalgia]. Med Clin (Barc) 2008;131:487–92. doi:10.1157/13127277.
